# Evolution in Composition of Kombucha Consortia over Three Consecutive Years in Production Context

**DOI:** 10.3390/foods11040614

**Published:** 2022-02-21

**Authors:** Perrine Mas, Thierry Tran, François Verdier, Antoine Martin, Hervé Alexandre, Cosette Grandvalet, Raphaëlle Tourdot-Maréchal

**Affiliations:** 1 UMR Procédés Alimentaires et Microbiologiques, Institut Agro Dijon, Université de Bourgogne Franche-Comté, Équipe Vin Alimentation Micro-organismes Stress (VAlMiS), 21000 Dijon, France; perrine.mas.pro@gmail.com (P.M.); rvalex@u-bourgogne.fr (H.A.); cosette.grandvalet@u-bourgogne.fr (C.G.); tourdot@u-bourgogne.fr (R.T.-M.); 2 Biomère, 14 rue Audubon, 75120 Paris, France; fverdier@jubiles.bio (F.V.); amartin@jubiles.bio (A.M.)

**Keywords:** kombucha, evolution, microbial composition, yeast, acetic acid bacteria

## Abstract

Kombucha is a traditional drink obtained from sugared tea that is transformed by a community of yeasts and bacteria. Its production has become industrialized, and the study of the microbial community’s evolution is needed to improve control over the process. This study followed the microbial composition of black and green kombucha tea over three consecutive years in a production facility using a culture-dependent method. Microorganisms were isolated and cultivated using selective agar media. The DNA of isolates was extracted, amplified using 26S and 16S PCR, and sequenced. Identities were obtained after a comparison to the NCBI database. *Dekkera/Brettanomyces bruxellensis*, *Hanseniaspora valbyensis* and *Saccharomyces cerevisiae* were the major yeast species, and the major bacterial genera were *Acetobacter* and *Liquorilactobacillus*. Results highlight the persistence of yeast species such as *B. bruxellensis* detected in 2019. Some yeasts species appeared to be sensitive towards stressful events, such as a hot period in 2019. However, they were resilient and isolated again in 2021, as was the case for *H. valbyensis*. Dominance of *B. bruxellensis* was clear in green and black tea kombucha, but proportions in yeasts varied depending on tea type and phase (liquid or biofilm). Composition in acetic acid and lactic acid bacteria showed a higher variability than yeasts with many changes in species over time.

## 1. Introduction

Kombucha is a drink obtained from sugared tea infusion through the activity of a symbiotic microbial community composed of yeasts (including *Saccharomyces cerevisiae*, *Torulaspora delbrueckii*, *Dekkera*/*Brettanomyces bruxellensis* (abridged *B. bruxellensis*) and bacteria, including acetic (mainly *Komagataeibacter*, *Gluconobacter* and *Acetobacter* genera) and sometimes lactic acid bacteria (from the genera *Lactobacillus* and *Liquorilactobacillus*) [1,2,3]. The production process occurs in two phases, the first in open conditions for acidification and another in closed conditions (after bottling for example) for natural carbonation [4]. Kombucha is a combination of several microbial activities, including alcoholic fermentation by yeasts and the oxidative metabolism by acetic bacteria [5]. Tea infusion creates the nitrogenous substrates, such as amino acids [6]. The sucrose added to the tea is hydrolyzed into glucose and fructose by the invertase activity of yeasts, which also produce ethanol through alcoholic fermentation. Acetic bacteria oxidize ethanol into acetic acid and glucose into gluconic acid [7]. The use of the yeast metabolites by bacteria raises the question of interactions between yeast and bacteria [8]. During the first phase, a cellulosic biofilm develops due to acetic acid bacteria and includes two environments for the development of microorganisms, which include a liquid phase, where the microorganisms have a planktonic lifestyle, and a solid phase, a cellulosic biofilm also called kombucha pellicle or SCOBY (Symbiotic Culture Of Bacteria and Yeasts) produced by acetic acid bacteria, where sessile cells remain [7,9].

As a result of these transformations, kombucha contains residual sugars, polyphenols, vitamins, organic acids (glucuronic, acetic, gluconic acids) resulting in pH value between 4.6 and 3.0 and low alcohol content (generally below 1% (*v*/*v*)) working as a barrier that is effective against microbial contamination [7,10]. Concentrations of each compound are dependent on process parameters and raw material [2,4]. To meet the increasing consumer demand, kombucha production has gradually become industrialized, leading to the development of many kombucha breweries of small to intermediate sizes. Therefore, this branch is relatively young in the process of business and technical structuring [11]. Moreover, kombucha microorganisms are poorly studied with complex interactions, whose study is relatively recent. Kombucha production meets difficulties related to reproducibility, the control of fermentations and therefore, the quality of the product, because of the complex nature of the consortia. Finally, the microbial community is likely to evolve as kombucha production progresses, with changes in the presence and proportions of species; however, field data about such evolution are lacking. Therefore, the long-term study of these microbial communities’ evolution appears crucial and has not been conducted before, to our knowledge. Indeed, changes in the populations could impact the functionality of the inoculum, which would lead to consequences in an industrial production process.

To determine and identify the genera and species composition of the kombucha microbial community used in a production unit, a culture-dependent approach has been chosen. Microorganisms were isolated using different selective or differential agar media, followed by the individual extraction, amplification, and sequencing of DNA through 26S and 16S, for yeasts and bacteria, respectively. Finally, the results obtained over three consecutive years (2019–2021) have been summarized and discussed.

## 2. Materials and Methods

### 2.1. Origin and Composition of Kombucha Samples

Green tea and black tea kombucha samples were obtained from the kombucha brewery Biomère (Paris, France) each year in January over three years in 2019–2021. The samples were kombucha liquid phase and biofilm with liquid sampled separately at the end of the acidification phase, before aromatization and bottling. The inoculation of new batches traditionally occurs by propagation [8]. The propagation lineages of green tea and black kombuchas originated from the same starter culture.

**Figure 1:**
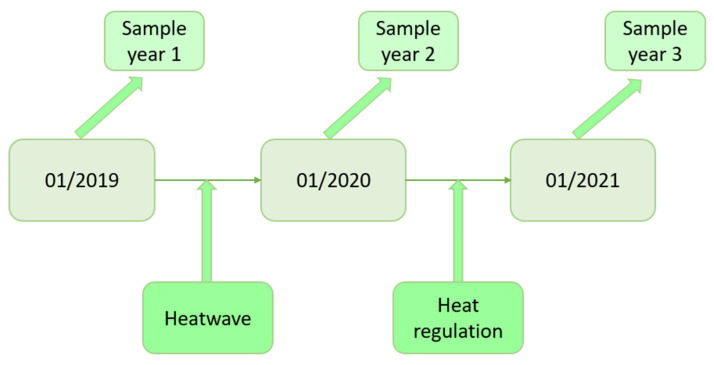
Timeline of experimentations and events at the production facility.

Kombucha of batch n-1 at the end of the acidification phase (approximately 7 days) was used to inoculate batch n at the rate of 12% (*v*/*v*). Approximately 20 propagations have been conducted each year. Production was carried out without temperature control, as no temperature regulation system was set up in the company before April 2020. Afterwards, temperature was maintained at around 25 ± 2 °C. It should be noted that a heat wave occurred during summer of year 2019, which occasionally led to production temperatures above 35 °C (Figure 1). Target chemical composition at the stage of sampling is the following: Brix° = 3.5 and pH = 3.5.

### 2.2. Isolation and Identification of Yeasts and Bacteria Using Culture-Depending Methods

#### 2.2.1. Sampling and Extraction of Microorganisms from the Biofilm

Liquid samples were directly used and diluted for plating whereas microorganisms needed to be extracted from the biofilm samples. The biofilm was gently removed and placed into a sterile Petri dish. Then, a punch sample of biofilm was cut with the help of a sterile beaker (3 cm diameter) and a sterile scalpel. The obtained sample was then rinsed twice with 10 mL of sterile physiological water. In parallel, an empty Petri dish filled with 10 mL of physiological water was weighted with and without the punch sample to calculate the fresh pellicle weight (average value of 1.6 g). Finally, the sample was placed in a sterile bag and homogenized for 30 min using a stomacher. 

#### 2.2.2. Determination of Biofilm Dry Weight

The determination of biofilm dry weight was performed on separate pieces of biofilm than the ones described in part 2.2.1. The biofilm punching and washing procedure was carried out in triplicates for each condition. Then, an aluminum cup was weighted with and without the punch to obtain its fresh weight. The dry weight was determined by drying at 102 °C for 24 h. Populations levels in the biofilm were expressed in CFU g^−1^ dry biofilm.

#### 2.2.3. Isolation of Microorganisms and Population Determination

Yeasts and bacteria were isolated using different agar media. The Wallerstein Laboratory medium of Thermo Fischer Scientific (Waltham, MA, USA) was used to isolate yeasts [12,13,14]. Bromocresol Green allowed for the macroscopic discrimination of colonies based on their appearance and color. Different media were used to isolate bacteria with different growth requirements. Mannitol agar medium with aerobic incubation was used to isolate acetic acid bacteria specifically [15]. De Man Rogosa and Sharpe (MRS) medium (pH 6.2) from Condalab (Madrid, Spain) in aerobic incubation was used to isolate lactobacilli. LAC (pH 5.1) [16] and M17 (pH 6.9; only used in 2020 and 2021) agar media with anaerobic incubation were used to isolate anaerobe lactic-acid bacteria, specifically with different nutritional requirements [17]. No unspecific agar medium was used to focus the description on yeast and bacteria species. All reagents used in culture media come from Merck (Darmstadt, Germany) if no details are given. The compositions of the agar media are provided in Appendix A. 

For each agar medium, 100 μL of each decimal dilution in physiological water was spread over the entire surface of the plate. Each plate counting was performed in triplicates. For liquid-phase samples, dilutions ranged from 10^−2^ to 10^−4^ and from 10^−2^ to 10^−5^ for biofilm samples. The detection limit was 1.10^3^ CFU mL^−1^. WL agar media were incubated for 72 h at 28 °C. The other agar media were incubated for 48 h at 28 °C.

#### 2.2.4. Macroscopic and Microscopic Examinations

Colony and cell morphotyping of yeasts and bacteria were carried out by macroscopic observation on agar plate and using an EVOS^®^ microscope from Thermo Fisher (Carlsbad, CA, USA) with 400 to 1000 magnification. However, because of the more similar aspects among bacteria colonies and cells, the same way of characterization could not be achieved.

#### 2.2.5. Preparation of Isolates for Identification

Five yeast colonies per colony morphotype were picked from the WL agar media, inoculated each in 0.5 mL of Yeast Peptone Dextrose medium (YPD), then incubated 48 h at 28 °C in closed tube. Then, the sample was centrifuged to 14,500× *g* three minutes, at 15 °C and the yeast pellets were re-suspended in 50% (*v*/*v*) YPD, 20% (*v*/*v*) glycerol.

The process was similar for acetic acid bacteria. However, fifteen colonies were picked without morphological distinctions on MRS and five on Mannitol agar media to inoculate each 0.5 mL of Mannitol medium. Then, liquid cultures were incubated for 48 h at 28 °C without agitation in open tubes, protected by a clean piece of cloth. After incubation and centrifugation (14,500× *g*, three minutes at 15 °C), the cells were re-suspended in 50% (*v*/*v*) Mannitol medium, 20% (*v*/*v*) glycerol. All isolates were stored at −20 °C.

For lactic acid bacteria, fifteen colonies were picked without morphological distinctions on LAC as well as five on M17 (only in 2020 and 2021). Each colony was inoculated in 1.5 mL of LAC medium in closed tubes. Then, liquid cultures were incubated 48 h at 28 °C without agitation. Finally, after centrifugation (14,500× *g*, three minutes at 15 °C), the bacteria were resuspended in 50% (*v*/*v*) LAC medium containing 20% (*v*/*v*) glycerol.

#### 2.2.6. DNA Extraction

DNA extraction was carried out on each isolate using an InstaGeneTM Matrix kit (BioRad, Hercules, CA, USA). The process was the same for all samples. First, 40 µL of InstaGeneTM Matrix reagent was mixed with 5 µL of thawed cell suspension. This suspension was incubated at 56 °C for fifty minutes, then at 99 °C for eight minutes before cooling down to room temperature. Afterwards, 30 µL of milliQ water was added. Finally, the sample was vortexed for thirty seconds and then centrifuged at 2500× *g* 10 min, at 10 °C, to separate extracted DNA from cellular debris.

#### 2.2.7. Amplification

To achieve interspecific discrimination for yeasts, an amplification of the 26S region of ribosomal DNA (D1/D2 domain) by PCR was performed. Both NL1 (5′-GCATATCAATAAGCGGAGGAAAAG-3′) and NL4 (5′-GTCCGTGTTTCAAGACGG-3′) primers were used [18]. Two microliters of DNA previously extracted with the InstaGeneTM Matrix kit was added to 25μL of the PCR mix (1.8 mM MgCl_2_, 0.25 mM dNTPs, 1.25 μM of each primer, and 0.025U Taq polymerase (Promega Corp., Madison, WI, USA), then amplification was performed using a BioRad thermocycler (Hercules, CA, USA) as described in Esteve-Zarzoso et al. (1999) [19]. 

Amplified DNA samples were analyzed by capillary electrophoresis with a MultiNa MCE 202 (Shimadzu, France), at 37 °C for 75 s using the DNA-1000 kit (Shimadzu, France), and an internal size calibrator. The peaks were identified after excitation of a LED (470 nm) and then detection of fluorescence. The size of the amplified DNA fragments was calculated by the software using the gene ruler 50 pb DNA Ladder molecular weight marker (Thermoscientific, Waltham, MA, USA) as a reference. 

To achieve strain discrimination for bacteria, RAPD (Random Amplified Polymorphic DNA) PCR amplification was performed using the primer M13, (5′-GAGGGTGGCGGTTCT-3′) [20]. Two microliters of DNA previously extracted with the InstaGeneTM Matrix kit were added to 25 μL of PCR mix (4 mM MgCl_2_, 0.20 mM dNTPs, 4 μM of each primer, and 0.05U Taq polymerase). The amplification was performed using a thermocycler (Qbiogen, Illkirch Graffenstaden, France), as described in Reguant and Bordons (2003) [21].

After selecting samples representative of the diversity of acetic and lactic bacteria through capillary electrophoresis analysis, an amplification of 16S rRNA sequence was performed. Ribosomal DNA extracted from the selected group was amplified using the following primers: E517F (5′-GCCAGCAGCCGCGGTAA-3′) and E106R (5′CTCACGRCACGAGCTGACG-3′) [22]. The amplification was carried out under the same conditions as for yeast samples.

#### 2.2.8. Identification

Amplified DNA samples obtained through 26S and 16S PCR were sequenced according to the Sanger method by Genewiz^®^ (Leipzig, Germany). Sequencing was performed on both strands, using primers E517F and E106R for bacteria, and NL1 and NL4 for yeasts. Finally, the sequences obtained were analyzed using the Geneious R7 software (version 7.1.5), and the BLAST tool (basic alignment search), which, after comparison with the NCBI databases, returned the names of genera and species associated with the E-value (number of expected hits of similar quality) and percentage of pairwise identity. The validation of genus and species per sample was made based on the lowest E-value.

### 2.3. Statistical Analyses

The confidence intervals with α = 0.05 associated with average values (m) have been calculated with Excel (Microsoft 365), from technical repetitions (n = 3). Its calculation involves standard deviation (σ). The results are expressed as follows: $m\pm 1.96\left(\frac{\sigma }{\sqrt{n}}\right)$.

## 3. Results

### 3.1. Populations of Yeasts and Bacteria

Microbial populations were determined in green tea kombucha and black tea kombucha (Table 1 and Table 2). In the liquid, total yeasts populations were equivalent between green and black tea kombucha for every year. In contrast for the biofilm, they were higher in green tea kombucha, except in 2019. Total yeasts populations were significantly higher or equivalent in the biofilm than in the liquid phase. A decrease was observed between 2019 and 2020 in both the liquid and biofilm. They increased again between 2020 and 2021. Total yeasts populations were systemically inferior or equal to the highest bacterial population regardless of the medium and phase (liquid or biofilm). The only exception is for the black and green tea biofilms in 2019. 

A comparison of bacteria populations between the different agar media showed significant differences. This can be explained by selective growth. Mannitol agar media with aerobic incubation and LAC agar media with anaerobic incubation are associated with the growth of acetic acid and lactic acid bacteria, respectively. Lactic acid bacteria populations were higher in black than green tea in the liquid (Table 1 and Table 2). This was not observed for acetic acid bacteria. As for yeasts, both acetic acid and lactic acid bacteria populations were higher in the biofilm than in the liquid, except in 2019 for lactic acid bacteria (Table 1 and Table 2). However, it is worth noting that the comparison of the populations between the solid and liquid phases is submitted to biases. Indeed, the accuracy of the count for the biofilm samples depended on the quality of the cell extraction. In the liquid, populations of acetic and lactic acid bacteria populations underwent similar variations as the yeasts with a decrease between 2019 and 2020 and an increase between 2020 and 2021 (Table 1 and Table 2). This behavior was not systematic in the biofilm, which highlights the differences in the effect of planktonic and sessile lifestyles.

### 3.2. Identification of Microorganisms

#### PCR and Electrophoresis

In 2019, 120 yeast clones and 200 bacteria clones were isolated. In 2020, 55 yeasts clones and 195 bacteria clones were isolated. In 2021, 88 yeasts clones and 168 bacteria clones were isolated. For yeasts, 26S PCR was performed using NL1 and NL4 primers, and amplified DNA samples were sequenced. The capillary electrophoresis profiles of amplified DNA could be observed for each sample. For 2019–2021, 24, 11 and 6 isolates, respectively, were selected for DNA sequencing. For bacteria, RAPD PCR was carried out using the M13 primer. Each isolate possessed several bands, and their position formed a profile that was dependent on the strain. The comparison of electrophoretic bands profiles allowed for the formation of groups with common profiles, and a representative isolate was selected from each of these groups. Then, for 2019–2021, 22,19 and 14 representative isolates, respectively, were amplified by PCR 16S and sequenced. A comparison of the sequences to the NCBI database returned identities with e-values equal to zero or very close (≤1.10^−173^). For some acetic acid bacteria, two species were proposed, because the targeted sequence did not allow for their discrimination. The identification results are presented in Table 3. 

For the yeasts, *B. bruxellensis* was found in green and black tea kombucha in both liquid and biofilm every year. It was the only yeast species identified in 2020 in black tea kombucha, and it was identified along with *Candida californica* in green tea kombucha. *Hanseniaspora valbyensis* was also widely detected for both tea types in 2019 and 2021. *Saccharomyces cerevisiae* was detected in 2019 in black tea kombucha only, and in 2021 for both tea types. However, in 2019 the population level was low and highly variable among the replicates (Table 2). Species such as *Hanseniaspora opuntiae*, *Galactomyces geotrichum* and *Pichia aff. fermentans* were only found in the biofilm of black tea kombucha, in 2019. During the same year, *C. californica* and *Candida boidinii* were isolated from green tea kombucha biofilm. Finally, the species *Zygosaccharomyces florentinus* was detected for the first time in 2021.

Regarding bacteria, the genera *Acetobacter* has been widely identified in black and green tea kombuchas over the years, except in 2021 black tea kombucha. However, the presence of species was not consistent between the years or tea types, but *A. indonesiensis tropicalis* or *senegalensis* and *A. peroxydans* or *papayae* were identified more often than the other species. Other genera including *Gluconobacter*, *Gluconacetobacter* and *Komagataeibacter* were not specific to a year or a tea type. With regard to lactic acid bacteria, *Liquorilactobacillus* (formerly belonging to the genus *Lactobacillus* [3]) was the only lactic acid bacteria genus identified and it was present in all samples except in 2020 black tea kombucha. Continuity in species could be observed between 2020 and 2021 for *L. mali* for green tea kombucha and for *L. ghanensis* and *L. nagelii* in black tea.

It appears that the yeast species *H. opuntiae*, *G. geotrichum* and *P. aff. fermentans* were specific to the biofilm along with many acetic acid bacteria species (*A. okinawensis*, *A. aceti* and *K. rhaeticus*), whereas this was not the case for lactic acid bacteria. 

Overall, the loss of species diversity of yeasts in 2020 was not as marked in bacteria, where several different species were still found, under all conditions. Moreover, it seems that the diversity of the bacterial population was less impacted than that of the yeast population in the biofilm in black tea kombucha.

### 3.3. Macroscopic and Microscopic Examinations

**Figure 2:**
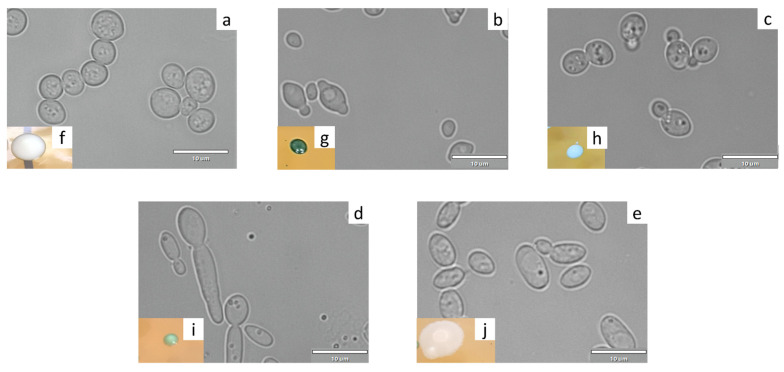
Cell and colony morphologies on WL agar medium of yeast identified in green and black tea kombuchas: (**a**,**f**) *Saccharomyces cerevisiae*, (**b**,**g**) *Hanseniaspora. valbyensis*, (**c**,**h**) *Zygosaccharomyces florentinus,* (**d**,**i**) *Dekkera/Brettanomyces bruxellensis*, (**e**,**j**) *Candida californica*.

To obtain more detail on populations according to genera and species, images were acquired using light microscopy to link the cellular aspect and colony morphotype on agar media to an identity. This was not possible with bacteria because of the very similar colony aspect overall. For yeasts, morphological differences could be easily observed, unlike in bacteria. Since the morphotypes stayed consistent on all the samples regardless of the year, location (liquid of biofilm) or tea type. Only an image of each species detected in 2021 is presented (Figure 2).

There was a visible diversity of morphology, with very round and opaque cells for *S. cerevisiae* (Figure 2a), apiculate yeasts in the case of *H. valbyensis* (Figure 2b), and very granular cells for the species *Z. florentinus* (Figure 2c). On agar medium, *B. bruxellensis* (Figure 2d) was associated with small white (1–2 mm diameter, sometimes light green), bulging, opaque colonies, with a diameter of less than 2 mm, which took 48 to 72 h to appear. Colonies of *H. valbyensis* (Figure 2b) were dark green, bulging, opaque, with a darker center. *S. cerevisiae* yeasts (Figure 2a) gave large white colonies (5–6 mm diameter, sometimes with a light green border), bulging and opaque. *Z. florentinus* (Figure 2c) was associated with very pale blue colonies, bulging, opaque, measuring between 1 and 2 mm. Finally, the colonies of *C. californica* (Figure 2e) were large (4–5 mm diameter), whitish, dull, opaque, and flat.

### 3.4. Proportions in Yeast Species

**Figure 3:**
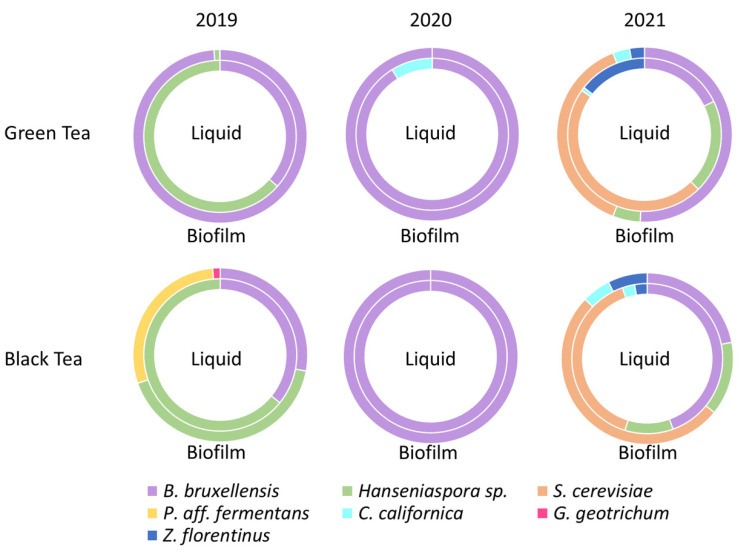
Proportions in yeasts species in green and black tea kombuchas liquid and biofilms samples over 3 years. Internal circles refer to the liquid phase and external circles to biofilm.

As yeasts have identifiable morphotypes, a morphotype count could be performed on WL medium to determine proportions. Figure 3 represents a translation of the “absolute” yeast populations shown in Table 1 and Table 2. The diversity within green tea kombucha has fluctuated over the three years. Indeed, it was observed that in 2019, the two genera *Hanseniaspora* and especially *Dekkera/Brettanomyces* were found in a major proportion in both phases, the genus *Candida* being in minority in SCOBY only (<1%) (Table 1). In 2020, the diversity decreased especially for the biofilm where only *B. bruxellensis* was detected, the liquid phase having preserved *C. californica* in smaller proportion (9%). On the other hand, the genus *Hanseniaspora* was not detected (Table 1). Finally, in 2021, diversity has been restored and was even higher than in 2019. The genera *Saccharomyces* and *Zygosaccharomyces* were detected for the first time in the green tea matrix, in important proportion for *S. cerevisiae* (47% in liquid phase) and the specie *H. valbyensis* was again present (Table 2). It was observed that the species *B. bruxellensis* was still represented, especially in the biofilm, where it represented 51% of the colonies (Figure 3). This species has been detected in an important proportion (even mostly) every year.

In black tea kombucha, the representation of different genera also fluctuated during the three years. In 2019, the genus *Hanseniaspora* and *B. bruxellensis* were the majority, respectively 64% and 36% for the liquid phase (Figure 3). In the biofilm, *Pichia* aff. *fermentans* was also well represented (29%), whereas *G. geotrichum* was a minority (<1%). Other genera were detected but their presence was very low (Table 2). In 2020, as in green tea, a massive drop in diversity was observed to the point that only *B. bruxellensis* was detected for each of the phases (Table 2). In January 2021, the diversity partly recovered as well in black tea kombucha, in which the species *H. valbyensis* was detected again and represented 10% and 14% of the colonies (Figure 3), but the other two genera (*Pichia* and *Galactomyces*) were not detected. Two new species were detected for black tea kombucha, which were *Zygosaccharomyces florentinus* and *Candida californica*, in minor proportions (Table 2).

A comparison of tea types showed that five yeast species were detected for the black tea matrix and three were detected for the green tea matrix in 2019. Conversely, in 2020, only the *B. bruxellensis* species was detected in both matrices, as well as *Candida* for green tea. Nevertheless, the diversity increased again in 2021, where five species were detected for each matrix, some of which were already present in 2019 and 2020, such as *B. bruxellensis* for example, or *Candida* (Table 1 and Table 2). Black tea kombucha had a greater diversity than green tea, especially in the biofilm. Regardless of population variations, the species found in 2021 were the same for both tea matrices (Table 1 and Table 2). Finally, the species *B. bruxellensis* was the most represented yeast species for the three years studied.

## 4. Discussion

An analysis of microbial populations and the identification of isolates in kombucha liquid and biofilm samples gave insights into the proportion of yeasts and bacteria species. Several species of yeasts have been identified for several years and in variable proportions (Figure 3): *B. bruxellensis, H.* *valbyensis, C. californica* and *S. cerevisiae*, which have already been identified in kombucha [2]. When comparing the 3 years, *B*. *bruxellensis* was found in all of the conditions (biofilm and liquid of the two teas) every year, which suggests its high adaptation to the sugared tea matrix. Moreover, the species *H*. *valbyensis* was found in every kombucha sample of 2019 and 2021. Thus, *B. bruxellensis* and *H. valbyensis* were the predominant yeast species in kombuchas according to recent studies [23,24]. Additionally, the proportions also varied from one year to the other. For example, a higher proportion of *H. valbyensis* was present in the two kombucha matrices in 2019 than in 2021(Figure 3). Dominance of the genus *Dekkera/Brettanomyces*, specifically *B. bruxellensis* has been reported along with the presence of the genera *Saccharomyces* and *Zygosaccharomyces* [10]. Additionally, the genus *Hanseniaspora* was sometimes found in greater or lesser proportions according to studies [10,23]. Other works reported the presence of the genera *Torulaspora, Starmerella* and *Zygotorulaspora,* which were not found in our samples [1,10,23,25]. The differences observed may be due to the specificities intrinsic to each kombucha consortia, but also to the fact that the studies cited used a metagenomic approach, which allowed for the detection of viable but nonculturable or dead microorganisms.

The most probable hypothesis for the loss of yeast diversity is a problem of thermal regulation within the place of production (a heat wave that took place during the summer of 2019; Figure 1), which would have negatively impacted some yeast species that are more sensitive to heat. The implementation of a temperature regulation of the premises between 2020 and 2021 would have made it possible to find a diversity for the samples of January 2021, with a modification of the species present and the representativeness. In all cases, the resilience of *B. bruxellensis* against the perturbation that caused the loss of diversity observed suggests high adaptability in this species, as it has been reported in wine [26]. The detection of species that were detected in 2019 and not in 2020, shows that they were not destroyed by the stressful event, but suggests remanence instead. If yeasts are more impacted by events and stresses related to production conditions, the functionality of the consortium for kombucha production will be strongly impacted, since yeasts’ metabolism mainly impacts the fermentation kinetics [8].

Different species of bacteria could be identified, with a predominance of genera such as *Liquorilactobacillus* and *Acetobacter* (Table 3). The comparison of the three years showed different dynamics in comparison with the yeasts. Indeed, diversity was greater in bacteria, especially for the year 2020. However, even though all genera were present in January 2021, differences between species were noticeable between the three years. These genera were also reported in the literature, along species such as *O. oeni* and *A. okinawensis* in the green tea matrix, and also *L. nagelii* and *L. satsumensis* for the black tea matrix. The genera *Gluconobacter* and *Komagataeibacter* were also present, but with different species than those described by Coton et al. (2017). More broadly, the genera *Komagataeibacter*, *Liquorilactobacillus* and *Acetobacter* were found in significant proportions in many kombucha biofilms [23]

Many lactic acid bacteria species were identified with eight different species in 2021, while the previous two years have combined six (Table 3). This could be explained by a change in their representation in the community or by the introduction of the M17 medium in 2020, which allowed for the growth of these bacteria. It is worth noting that all of the identified lactic acid bacteria species were ex-*Lactobacillus* species that were re-classified as *Liquorilactobacillus* [3]. Therefore, the change in nomenclature was beneficial in this study since it allowed us to highlight a relationship between this new genus and kombucha and calls for further investigation. The problem of thermal regulation mentioned upstream did not have a great impact on the bacterial population. The decrease in total bacterial population and the loss of diversity were less intense. Results regarding bacteria highlight a higher resilience of the overall bacteria population, associated with a higher variability in genera and species in the consortia over time compared to yeasts. It is possible to hypothesize that the under-representation of the genera *Acetobacter* and *Gluconobacter* was due to the diversity specific to each kombucha consortia.

Finally, the culture-dependent approach did not allow for the detection of viable but nonculturable microorganisms, in opposition to metagenomics. Nevertheless, it more accurately reflected the active populations involved in the transformation of the matrix, by putting aside dead and viable nonculturable microorganisms. Moreover, the use of colony morphotyping allowed for a satisfactory degree of detail for the investigation of the yeast population at the genus and species level.

## 5. Conclusions

The microbial communities of kombucha have a complex nature because of their diversity, which could easily be observed under a microscope, more specifically for yeasts. Yet, this diversity could be investigated in detail thanks to the identification of different isolates using a culture-dependent method. The use of differential and selective agar media, morphotyping and sequencing provided an accessible insight into the microbial composition of the kombucha cultures in an industrial context.

Changes in representations of population could be observed qualitatively and quantitatively. *B. bruxellensis* was constant in presence and dominance in all samples while the presence and proportion of other yeasts varied. Differences in representations between the different tea types (black or green tea) and the different phases (biofilm or liquid) helped to conclude that there is a certain preference to environment depending on the microbial species [9,10,27]. Stressful events such as heat waves in absence of temperature control during production could be linked to changes in the proportions and diversity within the consortium, especially in the case of the yeasts’ community, which appears to be more sensitive to thermal stress than bacteria. However, the remanence of certain species such as *Hanseniaspora valbyensis* could be observed. Composition in bacterial species was very variable but *Acetobacter* ad *Liquorilactobacillus* were the main genera identified over the three years. Therefore, it would be interesting to study the stress response of these microbial communities, and the effects on manufacturing processes on the final sensory quality, to increase the control over kombucha production.

## Figures and Tables

**Table 1:** Populations in yeasts and bacteria in green tea kombucha liquid and biofilm samples over three years (expressed as: average value ± confidence interval with α = 0.05). nd = not determined.

Green Tea Kombucha	January 2019		January 2020		January 2021	
	Liquid (CFU mL^−1^)	Biofilm (CFU g^−1^ Dry Mass)	Liquid (CFU mL^−1^)	Biofilm (CFU g^−1^ Dry Mass)	Liquid (CFU mL^−1^)	Biofilm (CFU g^−1^ Dry Mass)
Yeasts						
Wallerstein Lab nutrient (WL)	1.66 × 10^6^ ±3.39 × 10^5^	1.03 × 10^8^ ±2.43 × 10^5^	3.40 × 10^4^ ±1.72 × 10^4^	1.60 × 10^6^ ±1.55 × 10^3^	9.83 × 10^6^ ±4.27 × 10^6^	5.70 × 10^7^ ±4.53 × 10^6^
*Dekkera/Brettanomyces bruxellensis*	6.03 × 10^5^ ±1.64 × 10^5^	1.02 × 10^8^ ±3.16 × 10^6^	3.10 × 10^4^ ±2.63 × 10^4^	1.60 × 10^6^ ±2.92 × 10^5^	7.05. × 0^6^ ±1.30 × 10^5^	1.95 × 10^7^ ±6.66 × 10^5^
*Hanseniaspora sp.*	1.05 × 10^6^ ±7.42 × 10^4^	1.09 × 10^6^ ±4.46 × 10^5^	<1 × 10^3^	<1 × 10^3^	7.80 × 10^6^ ±2.74 × 10^4^	1.95 × 10^6^ ±6.86 × 10^3^
*Saccharomyces cerevisiae*	<1 × 10^3^	<1 × 10^3^	<1 × 10^3^	<1 × 10^3^	1.86 × 10^7^ ±2.55 × 10^4^	1.47 × 10^7^ ±8.04 × 10^4^
*Zygosaccharomyces florentinus*	<1 × 10^3^	<1 × 10^3^	<1 × 10^3^	<1 × 10^3^	5.70 × 10^6^ ±3.14 × 10^4^	1.05 × 10^6^ ±2.94 × 10^3^
*Candida* sp.	<1 × 10^3^	3.68 × 10^4^ ±6.53 × 10^2^	3.00 × 10^3^ ±7.84 × 10^2^	<1 × 10^3^	3.00 × 10^5^ ±1.96 × 10^3^	1.20 × 10^6^ ±1.18 × 10^4^
Bacteria						
Mannitol	8.47 × 10^6^ ±6.23 × 10^5^	9.29 × 10^7^ ±1.16 × 10^6^	1.79 × 10^6^ ±4.33 × 10^5^	5.51 × 10^7^ ±3.90 × 10^4^	9.45 × 10^6^ ±1.95 × 10^6^	7.79 × 10^7^ ±4.22 × 10^6^
LAC	7.13 × 10^5^ ±3.28 × 10^5^	2.34 × 10^6^ ±3.60 × 10^5^	3.18 × 10^5^ ±3.44 × 10^4^	8.98 × 10^6^ ±3.10 × 10^3^	8.33 × 10^6^ ±1.27 × 10^7^	5.18 × 10^8^ ±1.59 × 10^7^
MRS	8.67 × 10^6^ ±5.09 × 10^6^	1.15 × 10^6^ ±3.64 × 10^5^	2.23 ×10^5^ ±1.51 × 10^4^	8.98 × 10^6^ ±1.36 × 10^3^	1.19 × 10^6^ ±1.57 × 10^6^	8.57 × 10^7^ ±2.91 × 10^6^
M17	nd	nd	1.73 × 10^5^ ±4.09 × 10^4^	nd	1.74 × 10^6^ ±6.23 × 10^5^	1.66 × 10^8^ ±5.88 × 10^6^

**Table 2:** Populations in yeasts and bacteria in black tea kombucha liquid and biofilm samples over three years (expressed as: average value ± confidence interval with α = 0.05). nd = not determined.

Black Tea Kombucha	January 2019		January 2020		January 2021	
	Liquid (CFU mL^−1^)	Biofilm (CFU g^−1^ Dry Mass)	Liquid (CFU mL^−1^)	Biofilm (CFU g^−1^ Dry Mass)	Liquid (CFU mL^−1^)	Biofilm (CFU g^−1^ Dry Mass)
Yeasts						
Wallerstein Lab nutrient (WL)	1.26 × 10^6^ ±1.59 × 10^5^	>6.29 × 10^8^	4.51 × 10^4^ ±1.20 × 10^4^	4.70 × 10^5^ ±1.08 × 10^3^	9.50 × 10^6^ ±2.45 × 10^6^	2.78 × 10^7^ ±1.14 × 10^6^
*Dekkera/Brettanomyces bruxellensis*	4.50 × 10^5^ ±2.35 × 10^5^	>1.76 × 10^8^	4.51 × 10^4^ ±1.47 × 10^3^	4.70 × 10^5^ ±1.63 × 10^4^	1.74 × 10^7^ ±2.20 × 10^5^	9.75 × 10^6^ ±2.89 × 10^5^
*Hanseniaspora* sp.	8.07 × 10^5^ ±1.63 × 10^5^	2.63 × 10^8^ ±1.16 × 10^6^	<1 × 10^3^	<1 × 10^3^	4.05 × 10^6^ ±4.21 × 10^4^	6.15 × 10^6^ ±2.06 × 10^4^
*Saccharomyces cerevisiae*	66.7 ±98.0	5.52 × 10^4^ ±2.26 × 10^3^	<1 × 10^3^	<1 × 10^3^	1.56 × 10^7^ ±2.55 × 10^4^	2.28 × 10^7^ ±5.68 × 10^4^
*Pichia* aff. *Fermentans*	<1 × 10^3^	1.82 × 10^8^ ±2.79 × 10^5^	<1 × 10^3^	<1 × 10^3^	<1 × 10^3^	<1 × 10^3^
*Zygosaccharomyces florentinus*	<1 × 10^3^	<1 × 10^3^	<1 × 10^3^	<1 × 10^3^	1.05 × 10^6^ ±1.27 × 10^4^	3.30 × 10^6^ ±2.74 × 10^4^
*Galactomyces. Geotrichum*	<1 × 10^3^	8.81 × 10^6^ ±1.13 × 10^5^	<1 × 10^3^	<1 × 10^3^	<1 × 10^3^	<1 × 10^3^
*Candida* sp.	<1 × 10^3^	<1 × 10^3^	<1 × 10^3^	<1 × 10^3^	1.05 × 10^6^ ±2.94 × 10^3^	2.40 × 10^6^ ±3.92 × 10^3^
Bacteria						
Mannitol	5.73 × 10^6^ ±1.31 × 10^5^	>1.76 × 10^8^	1.97 × 10^5^ ±2.94 × 10^3^	2.13 × 10^8^ ±2.65 × 10^2^	8.47 × 10^6^ ±6.23 × 10^5^	4.75 × 10^7^ ±2.72 × 10^6^
LAC	5.43 × 10^7^ ±2.83 × 10^7^	3.47 × 10^7^ ±6.63 × 10^5^	1.92 × 10^6^ ±5.37 × 10^4^	3.22 × 10^7^ ±4.83 × 10^3^	6.31 × 10^7^ ±1.46 × 10^6^	1.48 × 10^8^ ±7.37 × 10^6^
MRS	2.93 × 10^7^ ±4.71 × 10^6^	3.00 × 10^7^ ±1.11 × 10^6^	8.50 × 10^5^ ±1.35 × 10^4^	1.45 × 10^8^ ±1.21 × 10^3^	8.67 × 10^6^ ±5.09 × 10^6^	8.25 × 10^6^ ±1.77 × 10^6^
M17	nd	nd	2.62 × 10^5^ ±4.12 × 10^4^	2.22 × 10^6^ ±3.70 × 10^3^	4.28 × 10^6^ ±4.02 × 10^5^	5.99 × 10^7^ ±5.05 × 10^6^

**Table 3:** Identification of yeasts and bacteria in black and green tea kombucha liquid and biofilm samples over 3 years.

Species	Green Tea 2019	Green Tea 2020	Green Tea 2021	Black Tea 2019	Black Tea 2020	Black Tea 2021
Yeasts						
*Dekkera/Brettanomyces bruxellensis*	L + B	L + B	L + B	L + B	L + B	L + B
*Hanseniaspora valbyensis*	L + B	nd	nd	L + B	nd	L + B
*Hanseniaspora opuntiae*	nd	nd	nd	B	nd	nd
*Saccharomyces cerevisiae*	nd	nd	L + B	L + B	nd	L + B
*Pichia aff. fermentans*	nd	nd	nd	B	nd	nd
*Galactomyces geotrichum*	nd	nd	nd	B	nd	nd
*Zygosaccharomyces florentinus*	nd	nd	L + B	nd	nd	L + B
*Candida boidinii*	B	nd	nd	nd	nd	nd
*Candida californica*	B	L	L + B	nd	nd	L + B
Acetic acid bacteria						
*Acetobacter indonesiensis*	nd	nd	nd	L + B	nd	nd
*Acetobacter tropicalis* or *senegalis*	L + B	B	nd	nd	L	nd
*Acetobacter pasteurianus* or *cibinongensis*	nd	nd	L	nd	nd	nd
*Acetobacter okinawensis*	nd	nd	B	nd	nd	nd
*Acetobacter aceti*	nd	nd	B	nd	nd	nd
*Acetobacter peroxydans* or *papayae*	nd	nd	nd	L + B	B	nd
*Gluconobacter sp*	nd	nd	nd	nd	nd	L
*Gluconacetobacter liquefaciens*	L + B	nd	nd	nd	L	nd
*Gluconacetobacter takamatsuzukensis*	L + B	nd	nd	nd	nd	nd
*Komagataeibacter rhaeticus*	nd	nd	B	nd	nd	nd
*Komagataeibacter saccharivorans*	nd	nd	B	L + B	nd	nd
Lactic acid bacteria						
*Liquorilactobacillus ghanensis*	nd	nd	nd	nd	L	L + B
*Liquorilactobacillus hordei*	nd	B	nd	nd	nd	nd
*Liquorilactobacillus satsumensis* or *oeni*	nd	nd	nd	nd	nd	L + B
*Liquorilactobacillus mali*	nd	L	L	nd	L	nd
*Liquorilactobacillus nagelii*	L + B	nd	nd	nd	B	B

L = detected in the liquid, B = detected in the biofilm, L + B = detected in both phases, nd = not detected in any phase.

## Data Availability

Not applicable.

## Appendix A

Composition of liquid and agar media:

Yeast Peptone Dextrose (YPD): 2% (*m*/*v*) glucose, 0.5% (*m*/*v*) yeast extract, 1% (*m*/*v*) bactopeptone, 0.02% (*m*/*v*) chloramphenicol, 2% (*m*/*v*) agar pH 6.5.

Differential Wallerstein Laboratory nutrient agar medium from Thermoscientific (Waltham, MA, USA) (Hall, 1971; Pallmann et al., 2001): 0.4% (*m*/*v*) yeast extract, 0.05% (*m*/*v*) tryptone, 0.5% (*m*/*v*) glucose, 550 ppm (*m*/*v*) dihydrogen potassium phosphate, 425 ppm (*m*/*v*) potassium chloride, 125 ppm (*m*/*v*) calcium chloride, 125 ppm (*m*/*v*) iron (III) chloride, 2,5 ppm (*m*/*v*) magnesium sulphate, 220 ppm (*m*/*v*) bromocresol green, 0.02% (*m*/*v*) chloramphenicol, 0.05 ppm (*m*/*v*) natamycin (Delvocid^®^), 0.063 ppm (*m*/*v*) penicillin, 1.5% (*m*/*v*) agar, pH = 6.5.

Mannitol medium: 2.5% (*m*/*v*) mannitol, 1% (*m*/*v*) extrait de levure, 0.05 ppm (*m*/*v*) natamycine (Delvocid^®^), 0.063 ppm (*m*/*v*) penicillin, 2% (*m*/*v*) agar, pH 6.5.

MRS medium: 0.2% (*m*/*v*) dextrose, 0.1% (*m*/*v*) bactopeptone, 0.8% (*m*/*v*) beef extract, 0.5% (*m*/*v*) sodium acetate, 0.2% (*m*/*v*) yeast extract, 0.2% (*m*/*v*) dipotassium phosphate, 0.2% (*m*/*v*) ammonium citrate, 0.1% (*v*/*v*) Tween 80, 0.2% (*m*/*v*) magnesium sulphate, 0.005% (*m*/*v*) manganese sulphate, 0.05 ppm (*m*/*v*) natamycin (Delvocid^®^), 2% (*m*/*v*) agar, pH = 6.2.

LAC medium: 7.8% (*v*/*v*) white grape juice from Casino^®^, 3.3% (*m*/*v*) yeast extract, 0.06% (*v*/*v*) Tween 80, 0.008% (*m*/*v*) manganese sulphate, 0.05 ppm (*m*/*v*) natamycin (Delvocid^®^), 2.5% (*m*/*v*) agar, pH = 5.1.

M17 medium: 0.5% (*m*/*v*) tryptone, 0.5% (*m*/*v*) soy peptone, 0.5% (*m*/*v*) meat infusion, 0.25% (*m*/*v*) yeast extract, 0.05% (*m*/*v*) ascorbic acid, 0.025% (*m*/*v*) magnesium sulphate, 1.9% (*m*/*v*) disodium glycerophosphate, 1.1% (*m*/*v*) agar, pH = 6.9.
